# Deep phenotyping of insomnia: a multimodal assessment protocol

**DOI:** 10.3389/fpsyt.2025.1696593

**Published:** 2025-11-14

**Authors:** Yujin Lee, Seonmin Kim, Sujin Kim, Seung Pil Pack, Heon-Jeong Lee, Chul-Hyun Cho

**Affiliations:** 1 Department of Psychiatry, Kangnam Sacred Heart Hospital, Hallym University College of Medicine, Seoul, Republic of Korea; 2 Department of Medicine, Graduate School, Korea University, Seoul, Republic of Korea; 3 Department of Psychiatry, Korea University College of Medicine, Seoul, Republic of Korea; 4 Department of Biomedical Informatics, Korea University College of Medicine, Seoul, Republic of Korea; 5 Department of Biotechnology and Bioinformatics, Korea University, Sejong, Republic of Korea

**Keywords:** insomnia, sleep disorders, deep phenotyping, digital phenotyping, multimodal assessment, fNIRS, wearable devices

## Abstract

**Background:**

Insomnia is a highly prevalent condition, with heterogeneous clinical presentations and underlying mechanisms. Traditional assessment methods often fail to capture this complexity, thereby hindering the development of personalized treatments. This paper details a protocol for a study that employs a “deep phenotyping” approach to comprehensively characterize insomnia.

**Methods:**

This single-center prospective observational study recruited adults with insomnia and a parallel cohort of normal sleepers as the controls. Participants undergo a 4-week multimodal assessment. The assessment framework integrates four key data domains: (1) clinical assessment, involving self-reported data from a comprehensive battery of clinical and psychological questionnaires; (2) digital phenotyping, capturing real-world behavioral and physiological data through a wrist-worn wearable device and a smartphone application; (3) functional neuroimaging, using a baseline functional near-infrared spectroscopy (fNIRS) scan to measure prefrontal cortex activity; and (4) genomic and biomarker collection from blood samples for genomic and exploratory biomarker analyses. The study was conducted between March 2023 and October 2024, and all recruitment and data collection have been completed. The core analysis will employ advanced computational methods, including clustering and machine learning, to identify the distinct subtypes of insomnia.

**Discussion:**

By applying multivariate pattern analysis and machine learning techniques to this rich, integrated dataset, we aimed to identify distinct biopsychosocial phenotypes of insomnia. This deep phenotyping approach is expected to elucidate the heterogeneity of insomnia, paving the way for the development of targeted and personalized management strategies for individuals with sleep disorders.

**Clinical Trial Registration:**

Clinical Research Information Service KCT0009175; https://cris.nih.go.kr/cris/search/detailSearch.do?seq=26133

## 1 Introduction

Insomnia is one of the most prevalent and impactful sleep disorders, affecting approximately 10% of adults chronically and an additional 30-35% experiencing occasional symptoms (1, 2). It is characterized by difficulty in initiating or maintaining sleep despite adequate opportunity and is frequently accompanied by daytime impairment (3–5).

The etiology of insomnia is complex and multifactorial. The development and persistence of insomnia are influenced by a combination of genetic factors (6, 7), psychological stress, maladaptive sleep habits, and cognitive factors (1, 8, 9). Circadian rhythm disruption (10), shift work (11), and comorbid medical (12) and psychiatric disorders (13) are also well-established risk factors.

Moreover, recent neuroimaging studies have suggested that individuals with insomnia may exhibit structural and functional alterations (14) in the brain regions associated with arousal, emotion regulation, and cognitive control, although the findings remain somewhat inconsistent (15). In parallel, emerging genetic research has highlighted the dysregulation of circadian clock genes that contribute to the pathophysiology of insomnia. These multilayered causes and the inherent heterogeneity of insomnia suggest that approaching it as a single symptom-based disorder is a fundamental limitation.

Conventional studies on insomnia in clinical practice have relied predominantly on patient-reported outcomes, including sleep questionnaires, sleep diaries, and clinical interviews. While polysomnography (PSG) and actigraphy offer more objective measures of sleep, their application is often limited to brief observation periods or specialized research settings. Consequently, traditional assessment approaches, which primarily focus on subjective symptoms or single-night metrics, often fail to capture the complex multidimensional complex nature of insomnia (16).

Ecological momentary assessment provides valuable real-time symptom data but remains limited by its reliance on subjective reports and narrow physiological measures, lacking insight into brain function and the biological underpinnings of insomnia (17, 18). Recent advances in digital phenotyping have enabled large-scale, real-time monitoring of sleep. However, such approaches are limited by the extreme complexity and heterogeneity of datasets and lack of subjective assessments (19, 20). In conclusion, fragmented approaches have a fundamental limitation in fully understanding the complex biopsychosocial characteristics of insomnia and identifying individual specificities.

Therefore, a multimodal assessment approach, commonly referred to as “deep phenotyping,” is essential to comprehensively unravel the complex and heterogeneous nature of insomnia. This approach seeks to integrate multiple levels of information, including the patients’ subjective experiences, objective sleep-wake patterns, neurophysiological activity, and genetic predispositions. Although recent studies have begun to explore such integrative frameworks, the field is still in its early stages. To overcome these limitations, this study outlines a research protocol designed to apply a deep phenotyping strategy to insomnia. Specifically, it combines data from standardized clinical questionnaires, continuous digital phenotyping using wearable devices and smartphone applications, functional neuroimaging via portable functional near-infrared spectroscopy (fNIRS), and genomic and biomarker analyses. Through this comprehensive, multimodal approach, we aim to identify the distinct phenotypic subtypes of insomnia. Ultimately, exploring the underlying biological and neurophysiological mechanisms of these subtypes may lead to a more refined understanding of the disorder and inform the development of personalized mechanism-based treatment strategies.

## 2 Methods

### 2.1 Study design

**Figure 1:**
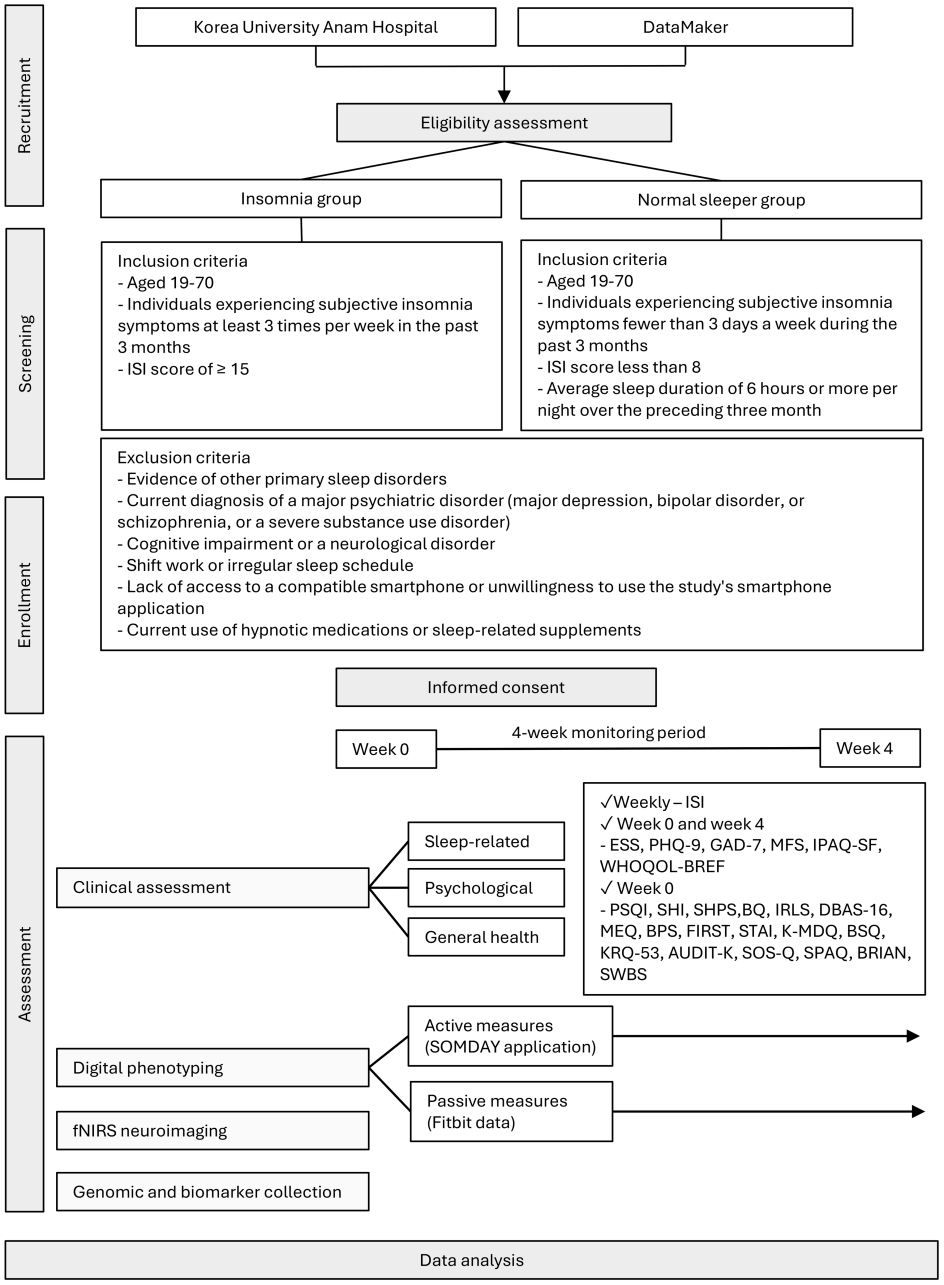
Protocol flow chart of the study fNIRS, functional near-infrared spectroscopy; ISI, Insomnia Severity Index; PSQI, Pittsburgh Sleep Quality Index; SHI, Sleep Health Index; SHPS, Sleep Hygiene and Practices Scale; BQ, Berlin Questionnaire; IRLS, International Restless Legs Syndrome Rating Scale; DBAS-16, Dysfunctional Beliefs and Attitudes about Sleep-16; MEQ, Morningness–Eveningness Questionnaire; BPS, Bedtime Procrastination Scale; FIRST, Ford Insomnia Response to Stress Test; STAI, State–Trait Anxiety Inventory; K-MDQ, Korean version of the Mood Disorder Questionnaire; BSQ, Body Sensations Questionnaire; KRQ-53, Korean Resilience Quotient-53; AUDIT, Alcohol Use Disorders Identification Test; SOS-Q, Smartphone Overuse Screening Questionnaire; SPAQ, Seasonal Pattern Assessment Questionnaire; BRIAN, Biological Rhythms Interview of Assessment in Neuropsychiatry; SWBS, Spiritual Well-Being Scale; ESS, Epworth Sleepiness Scale; PHQ-9, Patient Health Questionnaire-9; GAD-7, Generalized Anxiety Disorder-7; MFS, Multidimensional Fatigue Scale; IPAQ-SF, International Physical Activity Questionnaire–Short Form; WHOQOL-BREF, World Health Organization Quality of Life–BREF.

This study employed a single-center prospective observational design with two parallel cohorts, an insomnia group and a normal sleeper control group. The study was conducted at the Korea University Anam Hospital in Seoul, South Korea, and DataMaker Inc. in Daejeon, South Korea, between March 2023 and October 2024. The observational nature of the study allowed for the careful assessment and monitoring of participants’ sleep patterns, behaviors, and relevant physiological and biological parameters over a 4-week period. This duration was selected to capture a representative range of day-to-day fluctuations in sleep and daytime symptoms, while minimizing participant burden. A parallel-cohort design allows for the comparison of these measures between individuals with insomnia and those with normal sleep, enabling the identification of distinguishing features and potential subtypes of insomnia. The entire study consists of a baseline visit (week 0), a 4-week monitoring period (weeks 1-4), and an endpoint visit (week 4). An overview of this protocol is shown in **Figure 1**.

### 2.2 Participants

This study will recruit two parallel cohorts, an insomnia group and a normal sleeper control group. The inclusion and exclusion criteria for each cohort will be carefully defined to ensure a homogeneous sample and minimize confounding factors.

Participants in the insomnia group will be adults aged 19–70 years who report experiencing subjective insomnia symptoms, such as difficulty initiating sleep, difficulty maintaining sleep, or early morning awakenings, for at least three nights per week over the preceding three months. A score of 15 or higher on the Insomnia Severity Index (ISI) will be required to ensure that participants experience at least a moderate level of insomnia (21).

The normal sleeper control group will also consist of adults aged 19–70 years. To be included, these individuals must report experiencing insomnia symptoms less than three times per month, have an ISI score of less than 8, and report an average sleep duration of six hours or more per night over the preceding three months. Controls have no history of chronic insomnia or any major psychiatric illness and will also be required to comply with all the study procedures.

All participants in both groups are required to be willing to comply with all the study procedures, including the use of wearable devices and smartphone applications.

To minimize the influence of confounding factors, individuals meeting any of the following criteria were excluded from participation in either group: evidence of other primary sleep disorders such as untreated sleep apnea, narcolepsy, or other sleep-related breathing disorders; a current diagnosis of a major psychiatric disorder, including major depression, bipolar disorder, or schizophrenia, or a severe substance use disorder; the presence of cognitive impairment or a neurological disorder that could preclude valid self-report or compliance; participation in shift work or maintenance of an irregular sleep schedule within the past three months; and lack of access to a compatible smartphone or unwillingness to use the study’s smartphone application. The current use of hypnotic medications or supplements that have a significant effect on sleep was also an exclusion criterion.

### 2.3 Ethical considerations and informed consent

This study will be conducted in accordance with the principles of the Declaration of Helsinki. In accordance with the Bioethics and Safety Act and the Personal Information Protection Act (PIPA) of South Korea, all procedures concerning data handling and privacy protection were reviewed and approved by the IRB of Korea University Anam Hospital No. 2022AN0587). All participants in both cohorts will be required to provide written informed consent at the beginning of the study. The consent process will include a clear explanation of the study’s purpose, procedures, potential risks and benefits, data-handling procedures, and the voluntary nature of participation. Participants spent approximately 2 hours at enrollment for orientation and device setup, including the fNIRS assessment and blood sampling, and about 30 minutes for the final visit after 4 weeks. During the study, app-based daily assessments took about 1 minute, and weekly questionnaires required less than 5 minutes. All time requirements were explained to participants before enrollment. To ensure the protection of participants’ privacy, all personally identifiable information will be anonymized before data storage or analysis. Research data will be securely stored and accessible only to authorized personnel. Participants who complete the full study protocol, including the fNIRS session and blood sampling, will receive total compensation of 150,000 KRW, whereas those who complete all procedures except the fNIRS session and blood sampling will receive 100,000 KRW.

### 2.4 Clinical assessments

A comprehensive battery of validated clinical assessment instruments will be employed to characterize participants across multiple domains relevant to sleep, psychological functioning, and general health. These instruments are selected based on their psychometric properties, clinical relevance, and established uses in sleep research. Basic demographic information, including age, sex, work patterns, lifestyle factors, height, and weight are collected at the baseline. A detailed summary of all instruments, including the administration schedule and scoring, is provided in **Table 1**.

**Table 1:** Clinical assessment instruments.

Domain	Instrument (Abbreviation)	Administration Schedule	Scoring Range & Interpretation
Sleep-Related Assessments	Insomnia Severity Index (ISI)	Baseline, Weekly (via app), Week 4	7 items, 0–4 each (Total: 0–28). 0-7: No significant insomnia; 8-14: Mild; 15-21: Moderate; 22-28: Severe insomnia.
	Pittsburgh Sleep Quality Index (PSQI)	Baseline	19 items across 7 components (Total: 0–21). Global score >5 indicates poor sleep quality. Higher scores = worse sleep.
	Epworth Sleepiness Scale (ESS)	Baseline, Week 4	8 items, 0–3 each (Total: 0–24). >10 indicates excessive daytime sleepiness. Higher scores = greater sleepiness.
	Sleep Health Index (SHI)	Baseline	14 items across 3 domains (Total: 0–100). Higher scores indicate better sleep health. Assesses sleep quality, duration, and disordered sleep.
	Sleep Hygiene and Practices Scale (SHPS)	Baseline	30 items, 1–6 Likert scale (Total: 30–180). Higher scores indicate poorer sleep hygiene practices.
	Berlin Questionnaire (BQ)	Baseline	10 items across 3 categories. High risk if ≥2 categories positive. Screens for obstructive sleep apnea risk.
	International Restless Legs Syndrome Rating Scale (IRLS)	Baseline	10 items, 0–4 each (Total: 0–40). 0-10: Mild; 11-20: Moderate; 21-30: Severe; 31-40: Very severe RLS.
	Dysfunctional Beliefs and Attitudes about Sleep-16 (DBAS-16)	Baseline	16 items, 0–10 Likert scale. Mean score calculated (Range: 0–10). Higher scores = more dysfunctional sleep beliefs.
	Morningness-Eveningness Questionnaire (MEQ)	Baseline	19 items (Total: 16–86). Higher scores = morning preference; lower scores = evening preference. Determines chronotype.
	Bedtime Procrastination Scale (BPS)	Baseline	9 items, 1–5 Likert scale (Total: 9–45). Higher scores indicate greater bedtime procrastination tendency.
	Ford Insomnia Response to Stress Test (FIRST)	Baseline	9 items, 1–4 Likert scale (Total: 9–36). Higher scores = greater vulnerability to stress-induced insomnia.
Psychological and Mental Health Assessments	State-Trait Anxiety Inventory (STAI)	Baseline	40 items: 20 state + 20 trait items, 1–4 each (Range: 20–80 per subscale). Higher scores = greater anxiety.
	Patient Health Questionnaire-9 (PHQ-9)	Baseline, Week 4	9 items, 0–3 each (Total: 0–27). 5-9: Mild; 10-14: Moderate; 15-19: Moderately severe; 20-27: Severe depression.
	Generalized Anxiety Disorder-7 (GAD-7)	Baseline, Week 4	7 items, 0–3 each (Total: 0–21). 5-9: Mild; 10-14: Moderate; ≥15: Severe anxiety.
	Korean version of Mood Disorder Questionnaire (K-MDQ)	Baseline	13 yes/no items. Positive screen if ≥7 yes responses. Screens for bipolar spectrum disorders.
	Body Sensations Questionnaire (BSQ)	Baseline	17 items, 1–5 Likert scale. Mean score calculated. Higher scores = greater fear of autonomic sensations.
	Korean Resilience Quotient-53 (KRQ-53)	Baseline	53 items, 1–5 Likert scale across 3 domains. Higher scores indicate greater psychological resilience.
General Health and Lifestyle Assessments	Multidimensional Fatigue Scale (MFS)	Baseline, Week 4	18 items, 1–5 Likert scale (Total: 18–90). Assesses general, sleep/rest, and cognitive fatigue. Higher scores = greater fatigue.
	Alcohol Use Disorders Identification Test (AUDIT)	Baseline	10 items, 0–4 each (Total: 0–40). ≥8 indicates hazardous drinking; ≥20 suggests possible dependence.
	Smartphone Overuse Screening Questionnaire (SOS-Q)	Baseline	28 items, 1–4 point scale (Total: 28–112). Score ≥49 indicates high smartphone addiction risk.
	Seasonal Pattern Assessment Questionnaire (SPAQ)	Baseline	6 behavioral items, 0–4 each + severity rating. Global Seasonality Score ≥11 with marked problems suggests SAD.
	Biological Rhythms Interview of Assessment in Neuropsychiatry (BRIAN)	Baseline	18 items, 1–4 scale + 3 chronotype items (Total: 18–72). Higher scores indicate greater circadian rhythm disturbance.
	Spiritual Well-Being Scale (SWBS)	Baseline	20 items, 1–6 Likert scale (Total: 20–120). Comprises Religious (RWB) and Existential (EWB) subscales. Higher scores = better well-being.
	International Physical Activity Questionnaire-Short Form (IPAQ-SF)	Baseline, Week 4	7 items assessing walking, moderate, and vigorous activity. Results expressed as MET-minutes/week. Categorizes activity level.
	WHO Quality of Life-BREF (WHOQOL-BREF)	Baseline, Week 4	26 items across 4 domains, 1–5 scale. Domain scores transformed to 0–100 scale. Higher scores = better quality of life.

ISI, Insomnia Severity Index; PSQI, Pittsburgh Sleep Quality Index; ESS, Epworth Sleepiness Scale; SHI, Sleep Health Index; SHPS, Sleep Hygiene and Practices Scale; BQ, Berlin Questionnaire; IRLS, International Restless Legs Syndrome Rating Scale; DBAS-16, Dysfunctional Beliefs and Attitudes about Sleep-16; MEQ, Morningness-Eveningness Questionnaire; BPS, Bedtime Procrastination Scale; FIRST, Ford Insomnia Response to Stress Test; STAI, State-Trait Anxiety Inventory; PHQ-9, Patient Health Questionnaire-9; GAD-7, Generalized Anxiety Disorder-7; K-MDQ, Korean version of Mood Disorder Questionnaire; BSQ, Body Sensations Questionnaire; KRQ-53, Korean Resilience Quotient-53; MFS, Multidimensional Fatigue Scale; AUDIT-K, Alcohol Use Disorders Identification Test-Korea; AUDIT-C, Alcohol Use Disorders Identification Test-Consumption; SOS-Q, Smartphone Overuse Screening Questionnaire; SPAQ, Seasonal Pattern Assessment Questionnaire; K-BRIAN, Korean version of Biological Rhythms Interview of Assessment in Neuropsychiatry; SWBS, Spiritual Well-Being Scale; IPAQ-SF, International Physical Activity Questionnaire-Short Form; WHOQOL-BREF, World Health Organization Quality of Life-BREF; MET, Metabolic Equivalent of Task; SAD, Seasonal Affective Disorder; RWB, Religious Well-Being; EWB, Existential Well-Being.

#### 2.4.1 Sleep-related assessments

The sleep assessment battery was designed to capture multiple dimensions of sleep health and related behaviors that contribute to the pathophysiology of insomnia.

*Primary sleep measures*: The Insomnia Severity Index (ISI) (21, 22) serves as the primary outcome measure for insomnia severity, administered at screening for group allocation (≥15 for the insomnia group; <8 for controls) and weekly thereafter via the SOMDAY application. The Pittsburgh Sleep Quality Index (PSQI) (23, 24) assesses global sleep quality over the past month, whereas the Sleep Health Index (SHI) (25) provides a comprehensive measure of sleep health across multiple domains.

*Sleep disorder screening*: The Epworth Sleepiness Scale (ESS) (26, 27) measures daytime sleepiness, the Berlin Questionnaire (BQ) (28, 29) screens for obstructive sleep apnea risk, and the International Restless Legs Syndrome Rating Scale (IRLS) (30) assesses RLS (Restless Legs Syndrome) symptom severity.

*Sleep-related behaviors and cognitions*: Several instruments assess factors known to perpetuate insomnia. The Sleep Hygiene and Practices Scale (SHPS) (31, 32) evaluates sleep hygiene behaviors, whereas the Dysfunctional Beliefs and Attitudes about Sleep-16 (DBAS-16) (33) measures maladaptive sleep-related cognitions. The Morningness-Eveningness Questionnaire (MEQ) (34, 35) determines chronotype, Bedtime Procrastination Scale (BPS) (36) assesses bedtime delay behaviors, and Ford Insomnia Response to Stress Test (FIRST) (37) measures vulnerability to stress-induced sleep disturbance.

##### 2.4.1.1 Insomnia Severity Index

The ISI is a seven-item self-reported scale rated from 0 to 4 per item, with a total score ranging from 0 to 28. This scale assesses difficulty in initiating and maintaining sleep, early morning awakening, satisfaction with sleep, daytime functional impairment, impact on quality of life as observed by others, and worry about sleep problems. Scores of 0–7 indicate no clinically significant insomnia, 8–14 indicate mild insomnia, 15–21 indicate moderate clinical insomnia, and 22–28 indicate severe clinical insomnia. The ISI demonstrates a Cronbach’s α of 0.83, which reflects good internal consistency across diverse populations (38). In this study, the ISI is administered at screening to allocate participants (ISI ≥ 15 for the insomnia group; ISI < 8 for the control group), and thereafter, it was administered weekly to evaluate the subjective insomnia severity.

##### 2.4.1.2 Pittsburgh Sleep Quality Index

The PSQI is a 19-item self-report questionnaire that evaluates sleep quality across seven domains: subjective quality, latency, duration, efficiency, disturbances, medication use, and daytime dysfunction over the past month, yielding a global score ranging from 0 to 21. Each domain is scored from 0 to 3, with higher scores reflecting poorer sleep, and a global PSQI score >5 reliably distinguishes poor sleepers from good sleepers.

##### 2.4.1.3 Sleep Health Index

The SHI, developed by the National Sleep Foundation, is a validated self-report instrument designed to assess general sleep health in adults. It comprises 14 items grouped into three subdomains: sleep quality, sleep duration, and disordered sleep. Each subdomain contributes to a composite score ranging from 0 to 100, with higher scores indicating better sleep health.

##### 2.4.1.4 Epworth Sleepiness Scale

The ESS is a self-administered scale designed to measure average daytime sleepiness. This scale consists of eight items representing specific situations describing hypothetical situations such as sitting and reading, watching television, sitting inactive in a public space, riding as a passenger in a car for an hour without a break, lying down to rest in the afternoon, sitting and talking to someone, sitting quietly after a lunch without alcohol, and sitting in a car while stopped for a few minutes in traffic. Each item is rated on a 0–3 scale (0 = none, 1 = slight, 2 = moderate, 3 = high), resulting in a total score ranging from to 0-24.

##### 2.4.1.5 Berlin Questionnaire

The BQ is a 10-item self-report tool supplemented by height and weight data designed to assess obstructive sleep apnea (OSA) risk across three categories: snoring, witnessed apneas, daytime sleepiness and fatigue, and obesity/hypertension. A respondent is classified as high risk for OSA if two or more domains met the predefined positivity criteria. Categories 1 and 2 are considered positive if the total score is ≥2. Category 3 is considered positive if the participant has hypertension or BMI ≥25 kg/m^2^.

##### 2.4.1.6 Restless Legs Syndrome Rating Scale

The IRLS is a 10-item self-reported scale assessing core RLS symptoms, intensity and frequency, sleep disturbance, and impact on mood and daytime functioning. The total score ranges from 0 to 40, with higher scores indicating greater severity. Severity is classified as mild (0–10), moderate (11–20), severe (21–30), or very severe (31–40).

##### 2.4.1.7 Sleep Hygiene and Practices Scale

The SHPS is a 30-item self-report scale that captures a wide range of behaviors and environmental factors that may interfere with sleep. It is organized into four domains: arousal-related behaviors, sleep scheduling and timing, eating and drinking behaviors, and sleep environment. Each item is rated on a six-point Likert scale from 1 (never) to 6 (always), yielding a total score between 30 and 180, where higher scores indicate poorer sleep hygiene.

##### 2.4.1.8 Dysfunctional Beliefs and Attitudes about Sleep-16

The DBAS-16 assesses maladaptive sleep-related cognition using 16 items rated from 0 to 10 (“strongly disagree” to “strongly agree”). It evaluates four key domains: perceived consequences of insomnia, worry and helplessness about sleep, unrealistic sleep expectations, and beliefs regarding sleep medication. A global score is computed as the mean of all items (range 0–10), with higher total scores reflecting more dysfunctional sleep-related beliefs and greater cognitive contribution to sleep difficulties.

##### 2.4.1.9 Morningness-Eveningness Questionnaire

The MEQ is a 19-item self-report instrument uses to determine an individual’s chronotype based on preferred times for sleep/wake times, preferred times for activities, and subjective alertness. Items include a mixture of Likert-type and time-scale questions, each scored from 1 to 5, yielding a global score ranging from 16 to 86, with higher scores reflecting stronger morning preferences and lower scores reflecting stronger evening preferences.

###### 2.4.1.10 Bedtime Procrastination Scale

The BPS is a 9-item self-report measure designed to assess the tendency to delay bedtime without external justification, capturing a broad range of behaviors leading to insufficient sleep. Each item is rated on a five-point Likert scale from 1 (almost never) to 5 (almost always), resulting in total scores between 9 and 45, with higher scores indicating greater bedtime procrastination.

###### 2.4.1.11 Ford Insomnia Response to Stress Test

The FIRST is a 9-item self-report tool that assesses sleep reactivity, that is, the propensity to experience sleep disturbances in response to common stressful scenarios. Items describe situations such as work deadlines, social conflicts, or caffeine consumption, each rated on a four-point scale from 1 (not likely) to 4 (very likely), yielding a total score between 9 and 36, with higher scores indicating greater vulnerability to stress-induced insomnia.

#### 2.4.2 Psychological and mental health assessments

Given the strong bidirectional relationship between sleep and mental health, comprehensive psychological assessment is essential for phenotyping.

Current symptom severity is assessed using the Generalized Anxiety Disorder-7 (GAD-7) (39) and Patient Health Questionnaire-9 (PHQ-9) (40), both to be administered at the baseline and endpoint. The State-Trait Anxiety Inventory (STAI) (41) measures situational and dispositional anxiety. The Body Sensations Questionnaire (BSQ) (42) assesses anxiety sensitivity, particularly fear of autonomic sensations. The Korean version of the Mood Disorder Questionnaire (K-MDQ) (43, 44) screens for bipolar spectrum disorders using a simplified scoring algorithm (≥7 positive responses). The Korean Resilience Quotient-53 (KRQ-53) (45, 46) measures psychological resilience across emotion regulation, optimism, and problem-solving domains.

##### 2.4.2.1 State-Trait Anxiety Inventory

The STAI is a 40-item self-report inventory that measures two types of anxiety: state anxiety (how one feels “right now”) and trait anxiety (how one generally feels). Each subscale contains 20 items rated on a 4-point Likert scale from 1 (“almost never”) to 4 (“almost always”), yielding scores from 20 to 80 per subscale, with higher scores indicating greater anxiety levels.

##### 2.4.2.2 Generalized Anxiety Disorder-7

The GAD-7 is a brief, 7-item self-report scale designed to screen for generalized anxiety disorder and assess its severity over the prior two weeks. Each item is rated on a 4-point scale, ranging from 0 (“not at all”) to 3 (“nearly every day”), resulting in a total score of 0–21. The severity cutoffs are 5 (mild), 10 (moderate), and 15 (severe), with scores ≥ 10 warranting further evaluation.

##### 2.4.2.3 Patient Health Questionnaire-9

The PHQ-9 is a 9-item self-administered measure of depressive symptoms based directly on the DSM-IV criteria, covering the frequency of symptoms over the past two weeks. Items are scored from 0 (“not at all”) to 3 (“nearly every day”), yielding a total score of 0–27. Standard severity thresholds are 5–9 (mild), 10–14 (moderate), 15–19 (moderately severe), and 20–27 (severe).

##### 2.4.2.4 Body Sensations Questionnaire

The BSQ is a 17-item self-report inventory that assesses the degree of fear elicited by the common autonomic sensations associated with anxiety. Each item (e.g., heart palpitations, dizziness, shortness of breath) is rated on a 5-point Likert scale, ranging from 1 (“not frightened or worried”) to 5 (“extremely frightened or worried”), with higher averages reflecting greater tendencies toward catastrophic misinterpretation.

##### 2.4.2.5 Korean version of Mood Disorder Questionnaire

The Mood Disorder Questionnaire (MDQ) is a 13-item self-report screening tool for lifetime manic or hypomanic symptoms, indicative of bipolar spectrum disorders. It comprises three sections: (i) 13 yes/no items assessing lifetime manic/hypomanic symptoms; (ii) a question asking whether two or more endorsed symptoms occurred during the same period; and (iii) an item rating functional impairment on a scale from none to severe. In this study, we will use the validated Korean version of the Mood Disorder Questionnaire (K-MDQ), which applies a simplified scoring algorithm that considers only the section 1 symptom count. A cutoff of ≥7 yes responses indicates a positive screen, irrespective of symptom co-occurrence or impairment ratings.

##### 2.4.2.6 Korean Resilience Quotient-53

The KRQ-53 is a 53-item self-report instrument that measures psychological resilience across three core domains, emotion regulation, optimism, and problem-solving capacity, each represented by 17–18 items. Items use a 5-point Likert scale ranging from 1 (“strongly disagree”) to 5 (“strongly agree”), generating total and subscale scores that reflect one’s ability to adapt to stress and adversity.

#### 2.4.3 General health and lifestyle assessments

Additional instruments capture broader health and lifestyle factors that influence sleep and can confound study outcomes.

*Physical health and functioning*: The Multidimensional Fatigue Scale (MFS) (47) assesses fatigue across the general, sleep/rest, and cognitive domains. The WHO Quality of Life-BREF (WHOQOL-BREF) (48) measures perceived quality of life across four domains: physical, psychological, social, and environmental.

*Substance use and lifestyle factors*: The Alcohol Use Disorders Identification Test (AUDIT) (49, 50) screens for problematic alcohol use. The International Physical Activity Questionnaire-Short Form (IPAQ-SF) (51, 52) quantifies physical activity levels across walking, moderate, and vigorous-intensity activities.

*Circadian and seasonal factors*: The Biological Rhythms Interview of Assessment in Neuropsychiatry (BRIAN) (53, 54) assesses disturbances in circadian rhythms across sleep, social, activity, and eating domains. The Seasonal Pattern Assessment Questionnaire (SPAQ) (55) screens to assess seasonal mood variations.

*Technology use and spiritual well-being*: The Smartphone Overuse Screening Questionnaire (SOS-Q) (56) assesses smartphone addiction risk, whereas the Spiritual Well-Being Scale (SWBS) (57) measures religious and existential well-being.

##### 2.4.3.1 Multidimensional Fatigue Scale

The Multidimensional Fatigue Scale is an 18-item self-report questionnaire that evaluates three domains of fatigue—general fatigue, sleep/rest fatigue, and cognitive fatigue—using six items per domain. Each item is rated on a five-point Likert scale from 1 (“never”) to 5 (“almost always”), yielding subscale scores of 6–30 and a total score of 18–90, with higher scores indicating greater fatigue.

##### 2.4.3.2 Alcohol Use Disorders Identification Test

The Alcohol Use Disorders Identification Test (AUDIT) is a 10-item screening instrument that assesses alcohol consumption, dependence, and alcohol-related problems. Most items are rated 0–4; two items use a 0, 2, and 4 scoring format, with a total score between 0 and 40. A score of 8 or above suggests hazardous drinking that warrants further assessment, whereas higher cutoffs (e.g., ≥ 20) indicate probable alcohol dependence. This study will also use the Alcohol Use Disorders Identification Test-Consumption (AUDIT-C), a three-item consumption subscale of the AUDIT, to screen for hazardous drinking and possible alcohol use disorder. Each AUDIT-C item is scored 0–4, yielding a total score of 0–12. Sex-specific cut-offs are applied as follows: ≥4 for men and ≥3 for women.

##### 2.4.3.3 Smartphone Overuse Screening Questionnaire

The SOS-Q is a 28-item self-report screening questionnaire, with each item rated on a 4-point scale that identifies smartphone use habits and screens for smartphone addiction risk. The cutoff score is 49, and scores higher than 49 indicate a high risk of smartphone addiction.

##### 2.4.3.4 International Physical Activity Questionnaire–Short Form

The IPAQ-SF is a 7-item self-report measure of physical activity and sedentary behavior over the previous seven days. Participants report the number of days and average minutes per day they spent walking, performing moderate-intensity activities, and vigorous-intensity activities, as well as total sitting time. Responses are converted into metabolic equivalents-minutes per week to quantify the total activity volume.

##### 2.4.3.5 WHO Quality of Life–BREF

The WHOQOL-BREF is a 26-item self-report instrument derived from the WHOQOL-100 that assesses perceived quality of life across four domains: Physical Health, Psychological Health, Social Relationships, and Environment. Each item is rated on a five-point Likert scale, and raw domain scores are transformed to a 0–100 scale, where higher scores denote better quality of life. It includes two global items on overall quality of life and general health perception.

##### 2.4.3.6 Seasonal Pattern Assessment Questionnaire

The SPAQ is an 8-item self-report screener for seasonal affective changes in six behavioral and mood dimensions: sleep length, social activity, mood, weight, appetite, and energy. Each dimension is rated from 0 (“no change”) to 4 (“extremely marked change”), yielding a Global Seasonality Score (GSS) of 0–24. An additional severity item asks respondents to rate the degree of problems experienced due to these seasonal changes. A GSS ≥ 11 combined with moderate or marked problem severity indicates probable seasonal affective disorder.

##### 2.4.3.7 Biological Rhythms Interview of Assessment in Neuropsychiatry

BRIAN is an 18-item instrument designed to quantify circadian rhythm disturbances. It covers four primary domains (sleep patterns, social rhythms, activity levels, and eating behaviors) and includes three additional items for classifying an individual’s predominant rhythm (chronotype). Each item asks how often respondents experience disruption in maintaining a regular biological rhythm, rated on a four-point scale from 1 (“not at all”) to 4 (“very much”), resulting in a total score of 18–72, with higher scores indicating more severe circadian dysregulation.

##### 2.4.3.8 Spiritual Well-Being Scale

The Spiritual Well-Being Scale is a 20-item self-report measure of perceived spiritual quality of life, divided into two 10-item subscales: Religious Well-Being (RWB) and Existential Well-Being (EWB). Items are rated on a six-point Likert scale from 1 (“strongly disagree”) to 6 (“strongly agree”), yielding subscale scores of 10–60 and a total score of 20–120, with higher scores indicating greater spiritual well-being.

#### 2.4.4 Assessment schedule and administration

Clinical assessments are strategically scheduled to minimize the participant burden while capturing baseline characteristics and changes over the study period. Baseline assessments encompass a comprehensive battery of evaluations to establish a complete phenotype. Weekly assessments via the SOMDAY application focus on dynamic measures (e.g., ISI) to monitor symptom trajectories. Endpoint assessments (week 4) repeat the key measures (ESS, PHQ-9, GAD-7, MFS, IPAQ-SF, and WHOQOL-BREF) to evaluate changes in mental health, fatigue, physical activity, and quality of life.

### 2.5 Digital phenotyping

Digital phenotyping serves as a core methodological component of this study, using a wearable device and smartphone application for continuous monitoring of participants’ behavioral and physiological parameters. A custom-designed smartphone application, named “SOMDAY” (Lumanlab Inc., Seoul, Republic of Korea), is installed on the participants’ personal smartphones. The name combines “SOM” (from ‘somnus,’ the Latin word for sleep) and “DAY,” reflecting the key principle of circadian rhythms that optimizing daytime activities is crucial for improving nighttime sleep. The application is compatible with both the Android OS and iOS platforms.

Participants are provided with a wrist-worn wearable device (Fitbit Inspire 3, Fitbit Inc., USA) and are instructed to wear it throughout the 4-week study period, except for charging or if it causes significant discomfort. This integrated platform, consisting of a Fitbit device and a SOMDAY application, enables both passive and active data collection. The digital data to be collected for this study is categorized into four main domains: (1) sleep metrics, (2) activity data, (3) heart rate data, and (4) application-derived data.

The first three domains of data, which include sleep metrics, activity data, and heart rate data, are obtained passively from wearable devices and constitute the passive digital phenotyping component. The fourth domain, application data, is collected using a smartphone application and will represent the active digital phenotyping component. These data are processed to derive a comprehensive set of digital phenotypes for subsequent analyses.

#### 2.5.1 Passive digital phenotyping data collection

Fitbit devices continuously and passively collect objective data. Sleep metrics, including total sleep time, total awake time, sleep onset latency, sleep efficiency, and duration of sleep stage (light, deep, and REM sleep), are generated daily. Sleep stages are estimated using the Fitbit algorithm, based on a combination of movement and heart rate variability (HRV) patterns. Inactivity for approximately one hour is assumed to indicate sleep, while significant movements is interpreted as wakefulness. Total sleep time is calculated by subtracting the total awake time from the total inactive time.

The devices also collect physiological data, including continuous heart rate data (sampled at 5-minute intervals) and activity data, such as step counts and walking distance. Raw heart rate data will be utilized for cosinor analysis to derive circadian rhythm parameters. Step counts and walking distance, which are recorded as cumulative values, will be used to characterize circadian activity patterns. The data is automatically and wirelessly transmitted from the wearable device to the smartphone application and securely uploaded to a cloud-based research server.

#### 2.5.2 Active digital phenotyping data collection

The SOMDAY application will be used to actively collect self-reported data through a series of ecological momentary assessments (EMAs). To capture daily habits and experiences in a timely manner, participants will be prompted to log their daily entries every night at 9 PM. A daily sleep diary collects subjective information, including self-reported sleep duration, perceived sleep quality, and the number of awakenings. Additionally, participants receive daily ratings for their mood and stress levels. Moods are rated on a 7-point scale ranging from -3 (very bad) to +3 (very good), and stress levels are measured on a 4-point scale (0=none, 1=mild, 2=moderate, and 3=severe). The ratings for alcohol and caffeine consumption are collected daily, noting both the amount and the time of intake (morning, afternoon, or night). Detailed information on the time of day is crucial for applying a weighted scoring system during data processing to reflect the differential impact of these substances on the circadian rhythm. Information on smoking is collected as a daily log. Finally, brief questionnaires, such as the ISI, are administered weekly via the application to track longitudinal changes in insomnia severity and other related symptoms throughout the study period.

The SOMDAY application was used to actively collect self-reported data through a series of ecological momentary assessments (EMAs). The daily sleep diary was designed to be completed immediately after awakening, capturing self-reported sleep duration, perceived sleep quality, and the number of nocturnal awakenings. In addition, participants were prompted at 9:00 PM each day to report daily mood and stress levels, as well as alcohol and caffeine consumption.

Mood was rated on a 7-point scale ranging from −3 (very bad) to +3 (very good), and stress levels were rated on a 4-point scale (0 = none, 1 = mild, 2 = moderate, 3 = severe). Daily records of alcohol and caffeine intake included both the amount and the time of consumption (morning, afternoon, or night), allowing for weighted scoring to account for their differential effects on the circadian rhythm. Smoking behavior was also recorded as a daily log. Insomnia Severity Index (ISI) was administered weekly via the application to monitor longitudinal changes in insomnia severity and related symptoms throughout the study period.

#### 2.5.3 Data processing and feature generation

The raw digital data from the wearable devices and the SOMDAY smartphone application undergo a rigorous processing pipeline to generate a comprehensive set of digital phenotypes. This approach is not limited to a single analytical plan, and the processed data can be used for various advanced statistical and computational analyses, such as machine learning, to explore the complex relationships between different data modalities and identify meaningful patterns. The processing strategies for each data modality are meticulously designed to provide a rich dataset for subsequent analyses aimed at elucidating biopsychosocial phenotypes of sleep.

The periodic nature of sleep and activity is the key focus of our analysis. Raw data from wearable devices, including continuous heart rate and step counts, will be processed to extract a range of clinically relevant features. For continuous variables such as heart rate and step counts, cosinor analysis (58, 59) will be performed to characterize circadian rhythms. This will involve processing the data within 72-hour intervals to derive key circadian rhythm parameters, including midline estimating statistic of rhythm (MESOR), amplitude, and acrophase. MESOR represents the rhythm-adjusted mean and provides a robust measure of the average parameter level over time. Amplitude quantifies the extent of predictable variation within the circadian cycle, reflecting the strength of the rhythm. The acrophase indicates the timing of the peak value, which offers insight into circadian alignment or misalignment.

In addition, a range of other activity-related metrics will be calculated to provide a more detailed view of daily patterns. These will include the least active 5-hour period (L5), the most active 10-hour period (M10), interdaily stability (IS), and intradaily variability (IV) (60). L5 and M10 will be calculated using the moving average method to identify minimum and maximum activity periods, respectively. IS quantifies the day-to-day regularity of a rhythm, whereas IV measures rhythm fragmentation within a day. These features will be segregated by weekdays and weekends to capture distinct lifestyle patterns.

The raw, continuous heart rate data collected at 5-minute intervals will be a critical component for understanding circadian rhythmicity and autonomic function. We will apply cosinor analysis to these data, similar to the method used for step counts, to extract key rhythm parameters. Specifically, we will derive the MESOR, amplitude, and acrophase of the heart rate rhythm over a 72-hour window. The MESOR of the heart rate rhythm reflects the average heart rate, whereas the amplitude provides a measure of the magnitude of diurnal heart rate fluctuation, which can be an indicator of autonomic nervous system activity. The acrophase of the heart rate rhythm indicates the time of day when the heart rate is at its peak and is a key metric for assessing the phase of the circadian clock. These features will also be calculated separately for weekdays and weekends to account for differences in daily routines and their impact on physiological rhythms. Additionally, heart rate data collected during sleep will be used to derive the resting heart rate (RHR) as a proxy for autonomic nervous system activity, a measure known to be associated with arousal and stress. Heart rate data collected every 5 minutes from the Fitbit Inspire 3 were used to evaluate circadian rhythmicity and autonomic function. Because the device provides pulse rate variability derived from photoplethysmography(PPG) rather than true ECG based HRV, short term beat to beat analyses were not performed. Instead, resting heart rate during sleep and cosinor rhythm parameters were used to indirectly assess long term autonomic patterns. This approach allows for the characterization of autonomic rhythms in real world conditions.

Self-reported data collected via the SOMDAY application will undergo specific processing to reflect the differential impact of behavior on circadian regulation. A time-of-day weighted scoring system will be applied to substances such as alcohol and caffeine, based on their intake time (morning, afternoon, or night). For example, the disruptive potential of caffeine on sleep will be weighted more heavily for intake closer to bedtime. Similarly, stress levels and nap durations will be processed with weights to reflect their influence on circadian rhythms, a key focus of this study.

#### 2.5.4 Data adherence and privacy

The integration of the SOMDAY application with the Fitbit platform enables automatic synchronization of both active and passive data, allowing the research team to monitor participant compliance in real time. This system also allows participants to view their daily summaries, such as sleep and activity data, directly within the SOMDAY interface, thereby increasing self-awareness and motivation to adhere to the study protocol. In addition, an automated notification system sends encouragement messages when adherence for either data type falls below 50% within a given week, which further helps reduce participant fatigue and dropout. To ensure data security, all information collected through the SOMDAY application and Fitbit platform was encrypted during transmission and stored on an independent, access-restricted server maintained by the research team. Database encryption key management was implemented to prevent key leakage and unauthorized access. User access rights and activity logs were continuously monitored, and sensitive data were encrypted to prevent the exposure of personal identifiers.

### 2.6 Functional near-infrared spectroscopy neuroimaging

Prefrontal cortex activity is assessed using functional near-infrared spectroscopy (fNIRS), a non-invasive neuroimaging technique that measures hemodynamic responses associated with neural activation. fNIRS was selected because of its advantages in studying sleep disorders, including tolerance to head movement, silent operation, and suitability for participants who may have difficulty with traditional neuroimaging modalities.

#### 2.6.1 fNIRS system and setup

A portable multichannel fNIRS device (NIRSIT, OBELAB Inc., Seoul, Republic of Korea) is used to record the prefrontal hemodynamic responses. The system employs near-infrared light at dual wavelengths (760 and 850 nm) to measure the changes in oxygenated (HbO_2_) and deoxygenated (HbR) hemoglobin concentrations. The device features a flexible probe configuration optimized for prefrontal cortex coverage with source-detector separations of 30 mm to ensure adequate cortical sensitivity.

All fNIRS assessments are conducted at the Korea University Anam Hospital by trained research personnel, following standardized protocols. The session room maintained at a comfortable temperature and lighting conditions to minimize environmental confounding factors. The participants are seated comfortably with the fNIRS cap positioned according to the international 10–20 system, ensuring consistent probe placement across participants.

#### 2.6.2 Experimental protocol

The fNIRS assessment protocol consist of approximately 25 minutes of recording and structured as follows.

*Pre-task resting state (5 minutes)*: Participants maintain a relaxed state with their eyes open, fixating on a neutral stimulus (white cross on black background) to establish baseline prefrontal activity and functional connectivity patterns.

*Cognitive task battery (15 minutes)*: Two validated cognitive tasks are administered to probe distinct aspects of prefrontal function:

- *Visuospatial N-back task (8 minutes)*: This task assessed spatial working memory capacity, a cognitive domain frequently impaired in insomnia. Participants view a sequence of visual stimuli (stars) appearing at different locations in an 8-grid layout. They are required to respond when the current stimulus position matched the position from one, two, or three trials back (1-back, 2-back, 3-back conditions). Each difficulty level is presented in separate blocks (2 min each), with 1-minute rest periods between blocks. The performance metrics included accuracy, reaction time, and the d-prime sensitivity index.
- *Stroop color-word task (7 minutes)*: This task evaluated cognitive inhibition and attentional control. Participants view color words (red, blue, green, and yellow) displayed in either congruent or incongruent colors and are instructed to respond to the ink color while ignoring the word meaning. The task included three conditions: neutral (colored symbols), congruent (word and color matches), and incongruent (word and color mismatch). Each condition is presented for 2 min, with brief interblock intervals.

*Post-task resting state (5 minutes)*: A final resting-state period with eyes closed is recorded to assess post-task recovery and compare with pre-task connectivity patterns.

#### 2.6.3 Data acquisition parameters

The system monitor the signal quality in real time with automatic adjustments for probe contact optimization. The source-detector channel configurations cover bilateral prefrontal regions, including the dorsolateral prefrontal cortex (dlPFC), ventrolateral prefrontal cortex (vlPFC), and anterior prefrontal cortex (aPFC) regions.

Behavioral data from cognitive tasks is recorded synchronously with fNIRS signals, including response accuracy, reaction times, and response patterns. Task timing and event markers are automatically integrated with the fNIRS data stream to enable precise hemodynamic response analysis.

#### 2.6.4 Outcome measures and analysis framework

The primary fNIRS outcome measures were designed to capture task-related activation and intrinsic functional connectivity.

*Task-evoked activation*: Hemodynamic responses during cognitive tasks will be quantified as changes in the HbO_2_ concentration relative to the pre-task baseline. Task-specific activation patterns will be calculated for each cognitive domain (working memory and cognitive inhibition) and will be compared between insomnia and control groups.

*Resting-state functional connectivity*: Functional connectivity will be assessed by calculating Pearson correlations between HbO_2_ time series from different prefrontal regions during both pre- and post-task resting periods. Network topology measures, including node strength and clustering coefficients, will be derived to characterize the prefrontal network organization.

*Hemispheric asymmetry indices*: Lateralization of prefrontal function will be quantified using asymmetry indices by comparing left- and right-hemisphere activation and connectivity patterns. These measures provide insight into the potential hemispheric imbalance associated with insomnia.

*Cognitive performance-brain activity relationships*: Correlations between behavioral performance measures (accuracy, reaction time) and concurrent brain activation will be calculated to examine brain-behavior relationships and identify potential compensatory mechanisms in insomnia.

The fNIRS protocol will be specifically designed to be integrated with a broader deep phenotyping framework, with derived neural metrics to serve as features in computational analyses alongside clinical, digital, and genomic data to identify neurophysiological correlates of insomnia phenotypes.

### 2.7 Biological sample collection

Biological sample collection and genomic analysis are integral components of the deep phenotyping approach, providing insights into the genetic predisposition and molecular mechanisms underlying insomnia heterogeneity.

#### 2.7.1 Sample collection and processing

Venous blood samples (10 mL) are collected from all participants at the baseline visit using EDTA tubes to preserve nucleic acids. The blood samples are processed within 2 h of collection. The samples are centrifuged at 2000×g for 10 min at 4°C to separate the plasma from the cellular components. The buffy coat layer, which contains leukocytes rich in DNA and RNA, will be carefully extracted and aliquoted into cryovials. Plasma will be similarly aliquoted for potential biomarker analysis. All samples are labeled with unique study identification codes and stored at -80°C until analysis.

#### 2.7.2 DNA extraction and genotyping

High-quality genomic DNA will be extracted from buffy coat samples using the QIAamp DNA Blood Mini Kit (Qiagen, Germany) following the manufacturer’s protocol. DNA concentration and purity will be assessed using NanoDrop spectrophotometry, with samples meeting the quality criteria (260/280 ratio 1.8-2.0, DNA concentration ≥50 ng/μL) to proceed to genotyping.

Genome-wide genotyping will be performed using the Axiom PangenomiX Array (Thermo Fisher Scientific, USA), a high-density array capable of interrogating over 600,000 genetic variants, including single nucleotide polymorphisms (SNPs) and copy number variants. This array provides a comprehensive coverage of common and rare variants across the genome, including enhanced coverage of pharmacogenomic and clinically relevant loci.

#### 2.7.3 Polygenic risk score calculation

Primary genomic analysis will focus on calculating insomnia-specific Polygenic Risk Scores (PRS) rather than novel gene discovery. The PRS calculation will utilize summary statistics from the most recent and largest genome-wide association study (GWAS) of insomnia, leveraging data from over 1.3 million individuals.

PRS computation will follow established protocols:

- Quality control of genotype data including removal of variants with call rate <95%, minor allele frequency <1%, and Hardy-Weinberg equilibrium p-value <1×10^−6^
- Linkage disequilibrium clumping using European reference panels from the 1000 Genomes Project
- PRS calculation across multiple p-value thresholds (5×10^−8^, 0.001, 0.01, 0.05, 0.1, 0.5, and 1.0) to optimize predictive performance.
- Standardization of PRS values (mean=0, SD = 1) for interpretability

The resulting continuous PRS variable will quantify each participant’s genetic liability for insomnia, with higher scores indicating a greater genetic predisposition.

#### 2.7.4 Candidate gene expression analysis

Targeted gene expression analysis will be performed to examine the functional relevance of the key circadian and sleep-related genes. RNA will be extracted from buffy coat samples using the PAXgene Blood RNA Kit (PreAnalytiX, Switzerland), which preserved the *in vivo* gene expression profile at the time of blood collection.

Quantitative real-time PCR (qRT-PCR) will be performed using a focused panel of candidate genes.

*Core circadian clock genes*: *CLOCK, ARNTL/BMAL1, PER1, PER2, PER3, CRY1, CRY2, NPAS2*

*Clock-regulated genes*: *SIK1, SIK2, GSK3B (Glycogen Synthase Kinase 3 beta)*

*Neurotransmitter-related genes*: *COMT (Catechol-O-Methyltransferase)*

Gene expression levels will be normalized to housekeeping genes (*GAPDH* and *ACTB*) and will be expressed as fold-changes relative to a pooled reference sample.

#### 2.7.5 Data integration strategy

Genomic and biomarker data is designed for integration with clinical, digital phenotyping, and neuroimaging measures. The analytical framework conceptualized PRS as representing “genetic vulnerability,” gene expression patterns as “molecular state,” and biomarker levels as “physiological output” in the context of environmental and behavioral modulating factors captured through digital phenotyping.

This multi-omics integration approach will enable the investigation of gene-environment interactions, identification of molecular subtypes, and development of personalized risk prediction models. Specific analytical plans will include correlating PRS with digital phenotypes, examining gene expression patterns in relation to fNIRS findings, and testing whether genomic profiles modify the relationship between environmental factors and sleep outcome

### 2.8 Data analysis plan

The analytical strategy was designed as a multitiered approach, progressing from traditional statistical methods to advanced computational techniques to fully leverage the rich multimodal dataset. The analysis plan addressed three primary objectives: (1) characterizing the differences between insomnia and control groups, (2) identifying data-driven insomnia subtypes, and (3) developing predictive models for personalized sleep medicine.

#### 2.8.1 Preliminary data analysis and quality control

Data preprocessing: All datasets will undergo comprehensive quality-control procedures. Digital phenotyping data will be screened for outliers using interquartile ranges and temporal consistency checks. The clinical assessment data will be examined for completeness and consistency. fNIRS data undergo standard preprocessing, including motion artifact correction and signal quality assessment. Genomic data quality control will follow the established GWAS protocols.

*Missing data strategy*: Clinical and self-report variables will be handled using multiple imputation by chained equations (MICE) under the Missing at Random (MAR) assumption. For digital phenotyping data with systematic missingness (e.g., device non-wear periods), short-term gaps will be interpolated using Kalman smoothing to preserve temporal continuity, whereas longer missing periods will be excluded from time-series analyses. Sensitivity analyses will assess the impact of missing data assumptions on the primary findings.

*Descriptive statistics:* Comprehensive descriptive analyses will characterize both cohorts across all measurement domains. Continuous variables will be summarized using mean, standard deviation, median, and interquartile ranges. Categorical variables will be presented as frequencies and proportions. Data distributions will be assessed using histograms, Q-Q plots, and normality tests to inform subsequent analytical choices.

#### 2.8.2 Group comparison analyses

*Primary group comparisons*: Between-group differences (insomnia vs. controls) will be assessed using appropriate statistical tests based on the data distribution and measurement level. Independent samples t-tests (or Mann-Whitney U tests for non-normal distributions) will be used to compare continuous variables. Chi-square tests will evaluate categorical variables.

*Secondary analyses*: Subgroup comparisons will examine differences based on sleep disorder risk (e.g., RLS symptoms, sleep apnea risk) and demographic factors. Analysis of covariance (ANCOVA) will control for potential confounders, including age, sex, and comorbid conditions.

*Multiple comparisons*: Given the large number of variables, false discovery rate (FDR) correction using the Benjamini-Hochberg procedure will be applied to control for multiple testing. Both uncorrected and FDR-corrected p-values will be reported to balance the discovery and rigor.

#### 2.8.3 Correlation and regression analyses

*Bivariate associations*: Pearson correlations (or Spearman rank correlations for non-normal data) will be used to examine the relationships between subjective clinical measures (e.g., ISI scores) and objective digital phenotypes (e.g., sleep efficiency and circadian rhythm parameters). Correlation matrices will be visualized using heatmaps to identify patterns of association.

*Multivariate regression modeling*: Multiple linear and logistic regression models will be used to identify key predictors of sleep outcomes from the comprehensive digital phenotype set. Regularization techniques (LASSO, Ridge, and Elastic Net) will be employed to handle high-dimensional data and prevent overfitting. Model selection will utilize cross-validation procedures to optimize predictive performance while maintaining interpretability.

*Time-series analysis*: Longitudinal patterns in daily digital phenotypes will be analyzed using mixed-effects models to account for within-participant clustering. Time-varying covariates and random effects will capture individual trajectories and response heterogeneities.

#### 2.8.4 Unsupervised learning for phenotype discovery

*Clustering analysis*: Data-driven identification of insomnia subtypes will utilize multiple clustering algorithms, including k-means, hierarchical clustering, and Gaussian mixture models. Clustering will be performed using standardized digital phenotyping features, and the optimal cluster number to be determined using silhouette analysis, gap statistics, and clinical interpretability.

*Dimensionality reduction*: Principal component analysis (PCA) and t-distributed stochastic neighbor embedding (t-SNE) will reduce data dimensionality while preserving important patterns. Uniform manifold approximation and projection (UMAP) will provide an additional visualization of high-dimensional relationships.

*Cluster validation*: Discovered clusters will be validated using internal measures (silhouette width and Dunn index) and external validation through clinical outcomes and biomarker profiles. Stability analysis using bootstrap resampling will assess cluster robustness across different sample compositions.

#### 2.8.5 Supervised learning for prediction

*Model development*: Various machine learning algorithms will be employed for classification and regression tasks, including Random Forest, Extreme Gradient Boosting (XGBoost), Support Vector Machines, and neural networks. Tree-based models will be prioritized for their interpretability and robust performance using tabular healthcare data.

*Feature engineering*: Advanced feature engineering will create interaction terms, polynomial features, and domain-specific composite scores. Automated feature selection using recursive feature elimination and importance ranking will identify the optimal predictor sets.

*Model evaluation*: Predictive performance will be assessed using nested cross-validation to provide unbiased estimates. The classification tasks will use accuracy, sensitivity, specificity, area under the ROC curve (AUC), and precision-recall metrics. The regression tasks will employ mean absolute error, root mean square error, and R-squared values.

*Model interpretability*: Explainable AI techniques, particularly Shapley Additive explanation (SHAP) values, will quantify individual feature contributions to predictions. Partial dependence plots visualize feature effects, whereas permutation importance assesses global feature relevance.

#### 2.8.6 Multimodal data integration

*Data fusion strategies*: Multiple fusion approaches will be implemented, including early fusion (feature concatenation), late fusion (prediction averaging), and intermediate fusion (learned representations). Ensemble methods combine the predictions from domain-specific models to leverage complementary information.

*Deep learning approaches*: Neural network architectures designed for multimodal data will include autoencoders for dimensionality reduction and transformer models for integrating sequential data. These approaches will be applied judiciously, with sample size considerations and interpretability requirements guiding the implementation.

#### 2.8.7 Specialized analyses

*fNIRS data analysis*: Neuroimaging data analysis will follow established protocols using HOMER2/3 software. Statistical parametric mapping identifies task-related activation patterns, whereas functional connectivity analysis utilizes correlation and coherence measures. Group comparisons will be performed using general linear models with multiple comparison correction.

*Genomic data analysis*: Polygenic risk scores will be analyzed using linear models and population stratification controls. Gene expression data will employ differential expression analysis with multiple testing corrections. Pathway analysis using Gene Set Enrichment Analysis (GSEA) will identify biological mechanisms.

*Circadian analysis*: Cosinor analysis of physiological time series will employ nonlinear least-squares fitting to extract rhythm parameters. Population-mean cosinor analysis will test group-level rhythm differences, whereas individual cosinor analysis will characterize personal circadian profiles.

#### 2.8.8 Statistical software and reproducibility

All analyses will be conducted using R (version 4.3.0 or later) and Python (version 3.8 or later) with specific packages documented for reproducibility. Version control using Git will track all analysis codes. Computational notebooks (R Markdown and Jupyter) will provide transparent documentation of analytical decisions and results.

Statistical significance will be set at α = 0.05 for primary analyses, with appropriate corrections for multiple testing. Confidence intervals (95%) will be reported along with p-values to provide the effect size context. Sample size considerations were based on effect sizes from published insomnia studies and power analyses of machine-learning applications.

### 2.9 Sample size and power considerations

Participant recruitment for this deep phenotyping study has been completed in accordance with the original protocol design. The study employed a rolling, observational recruitment framework, with the goal of achieving a sample size sufficient for robust multimodal analyses across clinical, behavioral, physiological, and digital domains. Based on feasibility and methodological considerations, the protocol specified minimum recruitment targets of approximately 330 participants in total, including at least 240 individuals with insomnia and at least 80 good-sleeper controls.

These targets were established to ensure statistical adequacy for the key analytic aims while maintaining the feasibility of comprehensive multimodal data collection. Power estimations at the design stage indicated that, under this allocation (unequal groups, α = 0.05), the study would achieve about 80% power to detect standardized mean differences of Cohen’s d ≈ 0.36, which represents a small-to-moderate and clinically meaningful effect commonly observed in sleep research. For correlational analyses involving continuous variables, a total sample of this magnitude provides approximately 80% power to detect correlations of r ≈ 0.15–0.16, assuming complete data pairs. For predictive analyses, a balanced-class equivalent sample of this scale would permit area under the curve (AUC) estimates around 0.70 with a standard error of approximately 0.03, corresponding to a 95% confidence interval of roughly ±0.06. These calculations reflect the expected precision and effect sizes typical of multimodal insomnia research rather than formal hypothesis testing for a single endpoint.

In practice, 338 participants were enrolled, comprising 249 individuals with insomnia and 89 good-sleeper controls, consistent with the predefined recruitment objectives. Within the insomnia group, severity distribution according to the Insomnia Severity Index (ISI) was mild = 130, moderate = 101, severe = 18. Analyses involving ISI will therefore treat severity as a continuous variable or use pooled strata where appropriate, given limited sample sizes in the extreme categories. The analytic plan includes standard measures to ensure that statistical modeling remains commensurate with the available data, including internal cross-validation and regularization to reduce overfitting in multivariate and machine-learning models.

Overall, the final achieved sample meets and slightly exceeds the planned minimum targets, providing adequate statistical precision and methodological robustness for the multimodal analyses described in this protocol.

## 3 Discussion

This protocol presents a comprehensive and multimodal approach to the deep phenotyping of insomnia, a condition widely recognized for its clinical and etiological heterogeneity. Conventional assessment methods, often relying on demographic data analysis (61), self-reported subjective reports (62, 63), or single-night objective metrics, have shown limited reliability and poor concordance with perceived sleep quality (64, 65). Demographic data analyses and self-reported assessments (61–63) make it difficult to capture the complexity of insomnia. Therefore, our study aims to overcome these limitations by integrating data from clinical questionnaires, continuous digital phenotyping, functional neuroimaging, and genomics to comprehensively characterize the individual experiences of insomnia.

The primary strength and novelty of this protocol are its methodological integration. By combining continuous real-world data from wearables and smartphones with laboratory neurophysiological and biological measures, we can create a dataset of unprecedented depth and breadth. This approach intentionally bridges the gap between subjective, symptom-focused, and objective sleep measurement studies. Compared to single-night laboratory polysomnography, our wearable-based monitoring provides less detail on sleep architecture but offers far greater ecological validity by sampling multiple nights in the participant’s natural environment. This design enables the capture of the dynamic and fluctuating nature of insomnia, which static, single-time-point assessments often fail to do. However, this approach is not without its limitations. The sheer volume and complexity of multimodal data require sophisticated analytical techniques and careful management to avoid false discoveries. The intensive protocol may also impose a burden on participants, and the single-center design may limit the generalizability of the findings.

Implications of this deep phenotyping approach in sleep medicine are substantial. The primary object is to support a paradigm shift from undifferentiated care to personalized management. Tailoring insomnia treatments, such as cognitive behavioral therapy for insomnia (CBT-I) or pharmacotherapy, requires recognizing biologically distinct subtypes, including those characterized by physiological hyperarousal or circadian rhythm disruption (66–68).

More profoundly, the implications of this research approach extend beyond subtype discovery to both measurement and understanding of mental and sleep-related disorders. Continuous digital phenotyping replaces cross-sectional assessments with longitudinal measurements, enabling the development of predictive models that can forecast periods of heightened risk or detect early warning signs of relapse. Such models serve as the foundation for developing just-in-time adaptive interventions, where a digital platform can provide precise support when needed. Ultimately, this methodology aims to identify and validate novel digital biomarkers—objective, quantifiable indicators of the disease state derived from personal devices—which could substantially improve both clinical trials and routine patient monitoring, thereby contributing to precision sleep medicine. In conclusion, this protocol outlines a feasible and comprehensive strategy to characterize the complex phenotype of insomnia, with the potential to yield insights and tools that unidimensional approaches cannot provide.

Several limitations may affect the interpretation and generalizability of the findings of this deep phenotyping protocol. This study has limitations related to its single-center setting and sample composition. While a single-site setting allows for rigorous standardization of data collection, instrumentation, and participant monitoring—crucial for integrating multimodal measures such as digital phenotyping, neuroimaging, and genomics—it inherently limits representativeness. Convenience sampling and hospital-based recruitment may introduce selection bias, potentially overrepresenting individuals with more severe insomnia symptoms. Future multicenter and cross-cultural studies including more heterogeneous clinical populations will be necessary to validate and extend the generalizability of these findings. This observational design precludes causal inferences regarding the relationships between the identified phenotypes and clinical outcomes. The 4-week monitoring period may not capture the long-term trajectories or seasonal variations relevant to comprehensive phenotyping. Consumer-grade wearable devices have inherent accuracy limitations compared with PSG, particularly in the classification of sleep stages. Despite these constraints, wearable-based monitoring offers important advantages in terms of ecological validity, enabling the assessment of habitual sleep patterns and minimizing the first-night effect commonly observed in laboratory sleep studies. To address this limitation, future studies would benefit from incorporating a validation sub-study directly comparing Fitbit-derived sleep measures with simultaneous PSG recordings.

The wearable data in this study were sampled at 5-minute intervals and derived from photoplethysmography rather than ECG, which limits the temporal resolution and accuracy of short term autonomic measurements. Nevertheless, this approach provides valuable insight into long term physiological patterns in real world settings, and future studies using research grade devices with raw PPG or ECG signals could further improve measurement precision.

The genomic analyses in this study focused primarily on polygenic risk scores and candidate gene expression to establish a foundational molecular framework. However, the exclusion of epigenomic and proteomic biomarkers may limit the ability to capture dynamic molecular mechanisms underlying insomnia. Future studies incorporating multi-omics approaches, including DNA methylation and cytokine profiling, could enhance the biological depth and robustness of the phenotypic characterization. The fNIRS approach has limited spatial resolution compared to fMRI and focuses only on prefrontal cortex function. Although this configuration provides practical advantages in terms of ecological validity and participant comfort, it restricts the assessment of deeper or posterior brain regions, such as the thalamocortical circuits, that are relevant to the pathophysiology of insomnia. Future studies should consider applying complementary neuroimaging techniques, such as fMRI, to overcome these spatial constraints and achieve a more comprehensive understanding of the neurophysiological mechanisms underlying insomnia. The intensive digital phenotyping protocol may impose a significant participant burden, potentially affecting them over time, whereas technology literacy requirements may exclude certain demographic groups. The high-dimensional nature of multimodal data may increase the risk of overfitting in machine learning analyses. Although robust internal validation using nested cross-validation was implemented, the absence of independent external validation remains a limitation that should be addressed in future studies. Furthermore such high-dimensional, multimodal dataset increases the risk of multiple testing problems despite statistical corrections, and the sample size may limit the detection of rare phenotypic subtypes. The study setting differs from typical clinical environments, which may limit the direct translation to routine practice. The exclusion of major psychiatric disorders and restrictions to ages 19–70 years limit their applicability to key clinical populations.

Despite these limitations, this comprehensive deep phenotyping approach represents a significant methodological advancement that will inform future research and establish a foundational knowledge base regarding precision sleep medicine.
